# Supplementary material for: Differential privacy for eye tracking with temporal correlations
Source: PLoS One. 2021 Aug 17;16(8):e0255979. doi: 10.1371/journal.pone.0255979 (PMC8370645; doi:10.1371/journal.pone.0255979)
Supplement: S1 Table — (PDF) [file pone.0255979.s001.pdf]

S1 **Table. Document type classification accuracies in the MPIIDPEye dataset using differentially private eye movement features without majority voting.**

	Document type classification accuracies (k-NN|SVM|DT|RF)				
Method	$\epsilon = 0.48$	$\epsilon = 2.4$	$\epsilon = 4.8$	$\epsilon = 24$	$\epsilon = 48$
FPA	0.46|0.52|0.68|0.73	0.46|0.52|0.67|0.73	0.45|0.51|0.67|0.73	0.46|0.52|0.68|0.73	0.47|0.52|0.68|0.74
CFPA-32	0.34|0.35|0.36|0.38	0.34|0.35|0.36|0.38	0.34|0.36|0.36|0.38	0.39|0.44|0.38|0.42	0.47|0.53|0.44|0.49
CFPA-64	0.34|0.35|0.36|0.38	0.34|0.35|0.36|0.38	0.34|0.36|0.36|0.38	0.39|0.44|0.38|0.42	0.47|0.53|0.44|0.49
CFPA-128	0.34|0.34|0.36|0.39	0.34|0.34|0.36|0.39	0.34|0.34|0.36|0.39	0.38|0.42|0.37|0.42	0.46|0.51|0.43|0.48
DCFPA-32	0.36|0.35|0.36|0.37	0.36|0.34|0.35|0.37	0.35|0.34|0.36|0.37	0.36|0.35|0.35|0.37	0.35|0.34|0.36|0.38
DCFPA-64	0.38|0.37|0.35|0.37	0.37|0.35|0.35|0.37	0.37|0.36|0.35|0.37	0.37|0.36|0.35|0.37	0.37|0.36|0.35|0.37
DCFPA-128	0.40|0.38|0.36|0.38	0.39|0.37|0.35|0.37	0.41|0.39|0.35|0.38	0.38|0.37|0.35|0.38	0.39|0.37|0.35|0.38
