# Supplementary material for: Differential privacy for eye tracking with temporal correlations
Source: PLoS One. 2021 Aug 17;16(8):e0255979. doi: 10.1371/journal.pone.0255979 (PMC8370645; doi:10.1371/journal.pone.0255979)
Supplement: S2 Table — (PDF) [file pone.0255979.s002.pdf]

S2 **Table. Gender classification accuracies in the MPIIDPEye dataset using differentially private eye movement features without majority voting.**

	Gender classification accuracies (k-NN|SVM|DT|RF)				
Method	$\epsilon = 0.48$	$\epsilon = 2.4$	$\epsilon = 4.8$	$\epsilon = 24$	$\epsilon = 48$
FPA	0.48|0.42|0.48|0.45	0.48|0.42|0.47|0.44	0.47|0.41|0.47|0.45	0.47|0.41|0.48|0.44	0.48|0.43|0.48|0.45
CFPA-32	0.43|0.31|0.44|0.40	0.43|0.31|0.45|0.41	0.43|0.32|0.46|0.41	0.46|0.42|0.49|0.47	0.51|0.47|0.53|0.53
CFPA-64	0.44|0.35|0.45|0.40	0.44|0.35|0.45|0.41	0.44|0.35|0.46|0.42	0.46|0.43|0.49|0.47	0.51|0.48|0.54|0.53
CFPA-128	0.45|0.39|0.46|0.42	0.45|0.38|0.46|0.42	0.45|0.38|0.46|0.42	0.46|0.43|0.49|0.47	0.51|0.47|0.53|0.53
DCFPA-32	0.44|0.27|0.45|0.42	0.44|0.27|0.45|0.42	0.44|0.27|0.45|0.42	0.44|0.27|0.45|0.42	0.44|0.27|0.46|0.42
DCFPA-64	0.44|0.30|0.46|0.43	0.43|0.29|0.46|0.43	0.44|0.30|0.46|0.43	0.43|0.30|0.46|0.43	0.43|0.30|0.46|0.43
DCFPA-128	0.44|0.32|0.46|0.43	0.44|0.32|0.46|0.43	0.44|0.32|0.47|0.43	0.44|0.31|0.46|0.43	0.44|0.32|0.47|0.43
