# Supplementary material for: Differential privacy for eye tracking with temporal correlations
Source: PLoS One. 2021 Aug 17;16(8):e0255979. doi: 10.1371/journal.pone.0255979 (PMC8370645; doi:10.1371/journal.pone.0255979)
Supplement: S3 Table — (PDF) [file pone.0255979.s003.pdf]

S3 **Table. Person identification accuracies in the MPIIDPEye dataset using differentially private eye movement features without majority voting.**

	Person identification accuracies (k-NN|SVM|DT|RF)				
Method	$\epsilon = 0.48$	$\epsilon = 2.4$	$\epsilon = 4.8$	$\epsilon = 24$	$\epsilon = 48$
FPA	1|1|0.98|1	1|1|0.98|1	1|1|0.98|1	1|1|0.97|1	1|1|0.95|1
CFPA-32	0.09|0.11|0.16|0.16	0.08|0.11|0.16|0.17	0.09|0.11|0.17|0.17	0.12|0.15|0.18|0.21	0.18|0.21|0.23|0.27
CFPA-64	0.09|0.11|0.17|0.17	0.09|0.11|0.17|0.17	0.09|0.11|0.17|0.17	0.12|0.15|0.19|0.21	0.17|0.21|0.23|0.27
CFPA-128	0.11|0.13|0.17|0.18	0.11|0.13|0.17|0.18	0.11|0.13|0.17|0.18	0.13|0.16|0.18|0.20	0.18|0.20|0.21|0.25
DCFPA-32	0.09|0.10|0.15|0.16	0.09|0.11|0.14|0.16	0.09|0.11|0.14|0.16	0.09|0.11|0.14|0.16	0.09|0.11|0.15|0.16
DCFPA-64	0.09|0.10|0.13|0.15	0.09|0.10|0.13|0.15	0.09|0.10|0.13|0.15	0.09|0.10|0.13|0.15	0.09|0.10|0.13|0.15
DCFPA-128	0.08|0.09|0.12|0.13	0.08|0.09|0.11|0.13	0.08|0.09|0.11|0.13	0.08|0.09|0.12|0.13	0.08|0.09|0.11|0.13
