# Supplementary material for: Differential privacy for eye tracking with temporal correlations
Source: PLoS One. 2021 Aug 17;16(8):e0255979. doi: 10.1371/journal.pone.0255979 (PMC8370645; doi:10.1371/journal.pone.0255979)
Supplement: S4 Table — (PDF) [file pone.0255979.s004.pdf]

S4 **Table. Person identification classification accuracies in the MPIIPrivacEye dataset using differentially private eye movement features without majority voting.**

	Person identification classification accuracies (k-NN|SVM|DT|RF)				
Method	$\epsilon = 0.48$	$\epsilon = 2.4$	$\epsilon = 4.8$	$\epsilon = 24$	$\epsilon = 48$
FPA	1|1|0.99|1	1|1|0.99|1	1|1|0.99|1	1|1|0.97|1	1|1|0.94|1
CFPA-32	0.06|0.07|0.06|0.06	0.06|0.07|0.06|0.06	0.06|0.07|0.06|0.06	0.07|0.09|0.07|0.07	0.08|0.10|0.08|0.08
CFPA-64	0.06|0.07|0.06|0.06	0.06|0.07|0.06|0.06	0.06|0.07|0.06|0.06	0.07|0.09|0.07|0.07	0.08|0.10|0.07|0.08
CFPA-128	0.06|0.08|0.06|0.07	0.06|0.08|0.06|0.07	0.06|0.08|0.06|0.07	0.07|0.09|0.07|0.08	0.08|0.11|0.07|0.08
DCFPA-32	0.06|0.07|0.06|0.06	0.06|0.07|0.06|0.06	0.06|0.07|0.06|0.06	0.06|0.07|0.06|0.06	0.06|0.07|0.06|0.06
DCFPA-64	0.06|0.06|0.06|0.06	0.06|0.06|0.06|0.06	0.06|0.06|0.06|0.06	0.06|0.06|0.06|0.06	0.06|0.07|0.06|0.06
DCFPA-128	0.06|0.06|0.06|0.06	0.06|0.06|0.06|0.06	0.06|0.06|0.06|0.06	0.06|0.06|0.06|0.06	0.06|0.06|0.06|0.06
