# Supplementary material for: Differential privacy for eye tracking with temporal correlations
Source: PLoS One. 2021 Aug 17;16(8):e0255979. doi: 10.1371/journal.pone.0255979 (PMC8370645; doi:10.1371/journal.pone.0255979)
Supplement: Attachment — Submitted filename: Response to Reviewers.pdf [file pone.0255979.s005.pdf]

PONE-D-21-07774 Rebuttal Letter (Minor Revision)

Title: Differential Privacy for Eye Tracking with Temporal Correlations

We thank the Academic Editor and the Reviewers for evaluating our revised manuscript along with our rebuttal and for their helpful suggestions that helped to improve the manuscript. As further points are requested only by Reviewer 3, in our rebuttal, we address the points raised by the Reviewer 3. We hope that all concerns have been addressed in a satisfying way.

Response to Reviewer 3:

<u>1) The introduction does not fully explain the work in a high level manner adequately. Some important motivation for parts of the work are still missing, namely a) the explanation about the motivation of the utility analysis is missing b) the lack of motivation for using the epsilon-DP as a privacy metric, even though used in other works, the reader needs to know why such metric.</u>

Thank you very much for the suggestions.
a) The utility analysis based on the metric normalized mean square error (NMSE) shows the trend of divergence of noisy aggregated signals (i.e., differentially private signals) from the original signals and this metric is analytically trackable. We have stated this in the first paragraphs of “Results” and “Classification accuracy results” sections. To make this point clearer upfront, we have added this motivation to the last paragraph of “Introduction” section (Before “Previous research”) so that the reader can grasp the high level motivation before diving into technical details.

b) As requested, we have further explained the differential privacy and motivated its usage for eye movements in the second paragraph of the “Introduction” section. More technical details are already available in “Materials and methods” section.

<u>2) I recommend the authors to also think of a visual way to present their work, as it is not straightforward to understand how all the different privacy functions are used. I would also think of another way to present Algorithm 1, at the moment that algorithm seems to be not needed.</u>

Thank you very much for the suggestions. We have added a figure to visually depict FPA (instead of the algorithmic representation of it), since it is a fundamental approach that we use throughout the manuscript. In addition, we have added a visual representation combining the CFPA and DCFPA as suggested. These figures could be seen as Figures 1 and 2, respectively.

<u>3) In many answers in the response letter, the authors claim to have added more information in the discussion section. However, the additions made seem quite limited in terms of new information.</u>

Thank you very much for your request. We discuss our findings in “Discussion” section as well as providing their implications. While our “Results” section focuses on experimental

evaluations, their implications and comparisons with our initial expectations are discussed in "Discussion". To deepen the understanding based on our findings and comparisons, we have added further points to our "Discussion" section, especially to the first paragraph and strengthened the section by analyzing our findings in a more compact manner, especially by giving more details about the points mentioned in the previous revision and our previous rebuttal.

Thank you very much for your suggestions. We hope that all concerns have been addressed in a satisfying manner.
